# Calcination and ion substitution improve physicochemical and biological properties of nanohydroxyapatite for bone tissue engineering applications

**DOI:** 10.1038/s41598-023-42271-2

**Published:** 2023-09-16

**Authors:** Agata Kurzyk, Aleksandra Szwed-Georgiou, Joanna Pagacz, Agnieszka Antosik, Paulina Tymowicz-Grzyb, Anna Gerle, Piotr Szterner, Marcin Włodarczyk, Przemysław Płociński, Mateusz M. Urbaniak, Karolina Rudnicka, Monika Biernat

**Affiliations:** 1 Łukasiewicz Research Network, Institute of Ceramics and Building Materials, Cementowa 8 St., 31-983 Kraków, Poland; 2 Department of Immunology and Infectious Biology, Faculty of Biology and Environmental Protection, University of Lodz, 12/16 Banacha St., 90-237 Lodz, Poland; 3 Bio-Med-Chem Doctoral School, University of Lodz and Lodz Institutes of the Polish Academy of Sciences, 12/16 Banacha St., 90-237 Lodz, Poland

**Keywords:** Biomedical materials, Implants, Biomaterials - cells, Nanoparticles, Ceramics

## Abstract

Nanohydroxyapatite (nanoHAP) is widely used in bone regeneration, but there is a need to enhance its properties to provide stimuli for cell commitment and osteoconduction. This study examines the effect of calcination at 1200 °C on the physicochemical and biological properties of nanoHAP doped with magnesium (Mg^2+^), strontium (Sr^2+^), and zinc (Zn^2+^). A synergistic effect of dual modification on nanoHAP biological properties was investigated. The materials were characterized by X-ray diffraction (XRD), scanning electron microscopy (SEM), BET analysis, Fourier-transform spectroscopy, and thermal analysis methods. Furthermore, ion release tests and in vitro biological characterization, including cytocompatibility, reactive oxygen species production, osteoconductive potential and cell proliferation, were performed. The XRD results indicate that the ion substitution of nanoHAP has no effect on the apatite structure, and after calcination, β-tricalcium phosphate (β-TCP) is formed as an additional phase. SEM analysis showed that calcination induces the agglomeration of particles and changes in surface morphology. A decrease in the specific surface area and in the ion release rate was observed. Combining calcination and nanoHAP ion modification is beneficial for cell proliferation and osteoblast response and provide additional stimuli for cell commitment in bone regeneration.

## Introduction

Hydroxyapatite (HAP) is a naturally occurring mineral that forms the mineral phase of bone. Synthetic HAP plays a significant role in bone regeneration as it promotes the formation of new bone tissue by increasing cell adhesion enhancing cell differentiation and reducing inflammation^1^. Depending on the production method, the addition of HAP may result in a different morphology and microstructure, significantly affecting the interaction between the living tissue and the implant, the exchange of nutrients through the pore structures, osteointegration, and osteoinduction^2,3^.

Among several forms of apatites, there are biological apatites, found primarily in bones, teeth, and pathological tissues, such as kidney stones or bladder stones, and synthetic apatites. Organic apatites comprise calcium,phosphorus, trace elements and carbonate and citrate ions^4,5^. Chemical mineral composition of bones includes Sr (0–0.05 wt.%), Zn (0–39.00 ppm), Cr (0–0.33 ppm), Mn (0–0.17 ppm), Si (0–500 ppm), Co (0–0.025 ppm,) Cu (50–120 ppm), Li (0.001–0.06 ppm), F (0.03–0.10 wt.%), Cl (0.10–0.13 wt.%), Na (0.90–1.00 wt.%), and K (0.03–0.07 wt.%). Chemical minerals of dentin include Sr (0.04 wt%), Zn (173 ppm), Cr (2 ppm), Mn (2 ppm), Co (1 ppm), Cu (50–120 ppm), Li (0.001–0.06 ppm), F (0.07 wt.%), Cl (0.27 wt.%), Na (0.10 wt.%), and K (0.07 wt.%)^6,7^.

Synthetic hydroxyapatites contain varying calcium-phosphorus ratios. Purposeful Ca/P modifications can tailor apatite properties for specific applications in biomedicine. Ca/P ratios around 1.67 enhance material stability in the body. Calcium-deficient HA (with a ratio of 1.60) exhibits slightly higher bioactivity compared to stoichiometric HAP^8^. A Ca/P ratio exceeding 1.67 indicates calcium excess, potentially leading to modified biocompatibility, dissolution kinetics and mechanical properties. Additionally, calcination eliminates carbonates, aligning the material with standard pure hydroxyapatite^9^. Sintering at temperatures up to 850 °C can eliminate carbonate groups as well^10^. Hydroxyapatite can also be carbonated, resulting in types A and B; type B along with a mixed AB type, The latter holds importance for bone regeneration due to its biological similarity to organic apatites^11,12^.

To acquire multifunctional HAP particles, diverse HAP forms are developed by modifying synthesis methods and conditions^1–3^. Heating treatment conditions play a crucial role in influencing the properties of nanoparticles. Previous studies have indicated that higher temperature (above 1000 °C) treatment results in formation of certain phases, such as β-TCP and tetracalcium phosphate^13,14^. Thus, calcination serves a dual purpose, to aid in excess water and impurities removal from ceramics, and to actively facilitate phase formation^15^. Biological stimulation properties of HAP may be increased when additional modification are applied, e.g., ion substitution, by doping with small amounts of ions found in natural bone minerals, such as strontium, zinc, or magnesium^16,17^.

Strontium works antagonistically. Low doses of strontium stimulate osteoblast activity, reduce bone resorption, and inhibit apoptosis in osteoblasts, promoting bone formation and mineral metabolism^18^. Conversely, high concentrations can disrupt bone remodeling, hinder metabolism, and impede osteoclast apoptosis^19^. Thus, controlled strontium dosing is pivotal to achieve the desired benefits without adverse effects. Despite numerous studies on the topic, the mechanism of strontium’s impact on bone has not been fully recognized^20,21^.

The crucial element, zinc, is an essential cofactor for enzymes involved in synthesizing DNA, RNA, and proteins. It takes part in the repair of cell membranes and the regeneration of the musculoskeletal matrix^22^. Zinc also stimulates bone formation and inhibits bone resorption through osteoclasts, which is important for bone homeostasis and regeneration. Zinc’s positive effects are related to osteoblasts’ increased viability, osteogenic activity stimulation, bone healing, and antibacterial activity^23,24^. The optimal effects of zinc on osteoblasts occur within a narrow dose range (1–50 µM), and doses exceeding this range inhibit osteogenic activity^25^.

Magnesium is important for calcification and influences bone fragility^26^. Bones contain approximately 60% of the total body magnesium. In synthetic hydroxyapatite, magnesium content influences the crystal size and strength^27^. Moreover, magnesium plays an essential role in the induction of the proliferation of osteoblasts and fibroblasts, thereby promoting osteogenesis and bone regeneration^28^. Magnesium deficiency reduces bone stiffness, decreases osteoblast production, and promotes oxidative stress and subsequent bone loss, resulting in bone fragility^26^.

HAP modified by incorporating ions holds significant promise in addressing bone-related concerns, such as trauma or osteoporosis, by supporting bone regeneration and enhancing bone strength. For example, strontium (Sr^2+^), magnesium (Mg^2+^), or zinc (Zn^2+^) incorporation enhances HAP's bioactivity and its potential for bone healing. However, the medical application of ion-modified HAP requires further research to understand their impact on physicochemical and biological aspects and their intricate interplay. Moreover, the success of bone regeneration is contingent upon various influencing factors, including scaffold design, angiogenesis, growth factors, and immune response. As a result, achieving favorable outcomes in bone regeneration necessitates a comprehensive approach that accounts for the synergy of these factors.

This study aims to determine the effect of calcination at 1200 °C and ion modification on the physicochemical and biological properties of nanohydroxyapatite. Specifically, the study evaluates biocompatibility, reactive oxygen species activity, and osteoconduction of nanoHAP doped with magnesium (Mg^2+^), strontium (Sr^2+^), and zinc (Zn^2+^).

The innovative nature of this study lies in assessing the impact of dual modifications of nanoHAP as a candidate for material with biomedical applications. A simple method of producing modified nanoHAPs by ion doping based on precipitation from aqueous solutions, combined with innovative thermal processing, is presented. These novel synergistic dual modifications enhance the materials' potential to prompt cellular commitment, thereby supporting bone regeneration.

## Materials and methods

### Synthesis and modification of nanohydroxyapatite

Non-substituted (Ca_10_(PO_4_)_6_(OH)_2_) and substituted hydroxyapatites (Ca_10−x_Y_x_(PO_4_)_6_(OH)_2_ where Y is Mg, Sr or Zn) were prepared by the aqueous precipitation method from calcium hydroxide pure p.a. (Ca(OH)_2,_ Chempur) and 85% ortho-phosphoric acid, pure p.a. (H_3_PO_4_, Chempur) added dropwise over 45 min with constant stirring under alkaline conditions (pH = 11) by adding 25% ammonia solution (Chempur), if necessary. All reaction steps were performed at 50 °C. The solution was stirred (450–800 rpm) for approximately 2–3 h and then allowed to stand overnight at room temperature. The precipitate was rinsed 4–10 times with deionized water and centrifuged (3700 rpm for 2 min) until the excess ammonia solution was removed. Purified nanoHAP was dried at 90 °C for 12 h. Substituted hydroxyapatites were obtained by an analogous method, with the addition of precursors containing Mg^2+^ (Mg (NO_2_)_3_·6H_2_O; Chempur, pure p.a.), Sr^2+^ ((Sr(NO_3_)_2_; Sigma-Aldrich pure p.a), and Zn^2+^ (Zn(NO_2_)_3_·6H_2_O; Sigma-Aldrich, pure p.a.) to partially replace Ca^2+^ ions in the apatite structure. All compositions (Ca + X)/P molar ratio was 1.667, where X = Mg^2+^ or Sr^2+^ or Zn^2+^. The compositions of the synthesized samples and molar concentrations of the reactants used are given in Table 1.

**Table 1:** Molar concentrations of reactants and ion-calcium/phosphorus molar ratios.

Desired composition	Molar concentration of reactants (mol/L)					(Ca + X)/P molar ratio (X = Mg or Sr or Zn)
	Ca(OH)_2_	H_3_PO_4_	Mg(NO_3_)_2_*6H_2_O	Sr(NO_3_)_2_	Zn(NO_3_)_2_*6H_2_O	
Ca_10_ (PO_4_)_6_ (OH)_2_	1	0.6	**–**	**–**	**–**	1.67
Ca_9.9_ Mg_0.1_(PO_4_)_6_ (OH)_2_	0.9	0.6	0.1	**–**	**–**	1.67
Ca_9.9_ Sr_0.1_ (PO_4_)_6_ (OH)_2_	0.9	0.6	**–**	0.1	**–**	1.67
Ca_9.9_ Zn_0.1_ (PO_4_)_6_ (OH)_2_	0.99	0.6	**–**	**–**	0.1	1.67

The materials obtained were characterized, and the results were compared with pure HAP. A part of the nanoHAP powder was calcined at a temperature of 1200 °C for 1 h (conditions: heating at 10 °C/min to 1200 °C, holding at 1200 °C for 1 h, cooling at 10 °C/min to 20 °C and then air-cooling to room temperature), to ensure that possible traces of ammonium nitrate were eliminated from the synthesized nanoHAP materials. The properties of materials after the calcination process were compared with non-calcinated nanoparticles.

### Nanoparticles characterization

#### X-ray powder diffraction (XRD)

The phase composition of the synthesized powders was analyzed using the X-ray diffraction technique. The analyses were performed on a Bruker-AXS D8 DAVINCI diffractometer with a copper anode lamp in the Bragg–Brentano geometry. Diffractograms were recorded in the 2θ angular range from 5° to 120° (Cu Kα) at a measurement step of 0.01° and a measurement time 2 s/step. The crystalline phases were identified by comparing the registered diffractograms with the patterns in Crystallography Open Database (COD) using the DIFFRACplus EVA-SEARCH software. The lattice cells parameters, crystallite size and phase concentrations were calculated using the Rietveld method in Topas v5.0 software. To calculate crystallite size the LVol-IB was used which applies FWHM and integral breadth to give volume-weighted mean crystallite sizes.

#### Scanning electron microscopy—energy dispersive spectroscopy (SEM/EDS)

Scanning electron microscopy with field emission was used to define the microstructure (shape and grain size) of the obtained hydroxyapatites (Nova NanoSEM 200, FEI). The samples were covered with a conductive material (20 nm gold film) using a sputter coater (EM SCD500, Leica). The hydroxyapatite imaging was performed in high vacuum conditions using a secondary electron detector at 10 kV accelerating voltage and at a magnification of 100,000×. Energy dispersive spectroscopy analysis was carried out to confirm the ion substitution on the uncoated samples (Octane Elect EDS, Edax, combined with SEM). EDS analysis was performed in low vacuum conditions at 15 kV accelerating voltage.

#### Specific surface area determination (BET)

The Brunauer–Emmett–Teller (BET) method was used to assess a specific surface area of the samples with Gemini VII 2390t Micromeritics analyzer. The analysis was based on the determination of 9 points of nitrogen adsorption isotherm in the pressure range of 0.05–0.25 p/p°, where *p* and *p°* are the equilibrium and the saturation pressure of adsorbates at the liquid nitrogen temperature, respectively. Before N_2_ adsorption, the samples were degassed at 105 °C in the N_2_ atmosphere for 1 h to dry and purify the samples.

#### Fourier—transform infrared spectroscopy (FTIR)

The infrared spectra were recorded on Bruker TENSOR 27 instrument equipped with a DLaTGS detector. Powder samples were analyzed in the transmission mode with the following instrumental settings: wavenumber range of 400–4000/cm, number of scans 64, and spectral resolution 4/cm. Each sample was measured twice to check repeatability. The baseline correction procedure has been applied to the presented spectra with Opus 7.2 software.

#### Thermogravimetry differential thermal analysis (TG—DTA)

The thermal properties of the powdered materials were analyzed with a STA F3 449 Jupiter® Netzsch thermal analyzer. TG*–*DTA experiments were performed under a dynamic flow of argon (70 mL/min) to investigate the particular mass loss due to the presence of a modifier. The 10 − 12 mg samples were heated from 30 °C up to 1400 °C at a heating rate of 10 °C/min in an Al_2_O_3_ DTA pan with a lid and a hole. The measurements were repeated twice for each sample.

#### Ion release tests

The ion release was investigated by immersion of 1 g of each sample in 10 mL ultrapure water and further incubation. The incubation procedure was performed at 37 °C in a separated closed flask for each sample, and after 1, 7, 21, and 42 days, the collected supernatant was filtered through 0.45 µm paper filter. Determination of selected elements was performed with inductively coupled plasma optical emission spectrometry (ICP—OES) using a Shimadzu ICPE 9800 spectrometer. The calibration solutions were prepared from Merck ICP multielement standard XVI storage solution (for Ca, Mg, Zn, Sr). The quantification limits of the method for the cations were 0.08 mg/L for Ca, 0.09 mg/L for Mg, 0.10 mg/L for Zn, and 0.02 mg/L for Sr. The conducted analytical tests meet the ISO 11885:2009 requirements. All experiments were made in triplicate. Intergroup outcomes were compared between non-calcinated and calcinated samples for statistical significance using two-way ANOVA (analysis of variance) and Tukey's test.

## In vitro biological characterization

### Sterilization

Before the biological assays, all nanohydroxyapatite powders were sterilized by gamma radiation (35 kGy, _60_Co source) at the Institute of Applied Radiation Chemistry at the Lodz University of Technology (Lodz, Poland).

### Cell culture conditions

The L929 (CCL-1) mouse skin fibroblasts recommended by the International Standard Organization (ISO) for the biological evaluation of biomaterials for medical applications were obtained from the American Type Culture Collection (ATCC, Manassas, VA, USA). Fibroblasts were cultured in Roswell Park Memorial Institute (RPMI)-1640 medium supplemented with 10% heat-inactivated fetal bovine serum (FBS; HyCloneCytiva, Marlborough, MA, USA), penicillin (100 U/mL), and streptomycin (100 μg/mL) (Sigma-Aldrich, Darmstadt, Germany), in a humidified 5% CO_2_ atmosphere at 37 °C in cell culture incubator (Nuaire, Plymouth, MN, USA).

The human fetal osteoblastic cell line hFOB 1.19 (CRL-11372™) was obtained from the American Type Culture Collection (ATCC, Manassas, VA, USA). The cells were cultured in Dulbecco's Modified Eagle's Medium/Ham's Nutrient Mixture F12 without phenol red (1:1 DMEM/F12 Modified; Gibco; Thermo Fisher Scientific, Waltham, MA, USA) containing a 0.3 mg/mL geneticin (Sigma-Aldrich, Saint Louis, MO, USA) and supplemented with a 10% fetal bovine serum. The cell cultures were incubated at 34 °C in the incubator with a humidified air atmosphere containing 5% CO_2_.

The confluent (80–90%) cell monolayers were periodically subcultured using a 0.5% trypsin-0.5 mM ethylenediaminetetraacetic acid tetrasodium salt (EDTA) solution (Gibco, Thermo Fisher Scientific, Waltham, MA, USA). The cell viability was established using a trypan blue exclusion assay. If the cell’s viability exceeded 95%, the suspension was used for cell morphology and cell viability assessment.

### Direct contact cytotoxicity assay

To determine the influence of the prepared nanoHAP materials (at concentrations of 1 mg/mL, 5 mg/mL, and 10 mg/mL) on cell viability, in vitro cytosafety studies were carried out according to ISO-10993-5-2009 for testing components with potential application in biomedicine. Murine fibroblasts (L929, ATTC, Rockville, MD, USA) and human osteoblast cells (hFOB 1.19) followed by the MTT reduction assay were used as described previously^29^. Briefly, after the overnight incubation of the cells with the tested nanoHAP materials, 20 μL of MTT reagent (Sigma Aldrich) at a concentration of 5 mg/mL was introduced into each well and incubated for 4 h (5% CO_2_, 37 °C, > 90% humidity). In the next step, the plates were centrifuged (1200 rpm, 10 min), and the supernatants were removed and replaced with 200 μL DMSO per well. After one minute of incubation at room temperature with shaking, the absorbance was measured at 570 nm using the Multiskan EX reader (Thermo Scientific).

### Quantification of reactive oxygen species (ROS)

For measuring changes in reactive oxygen species (ROS) levels in hFOB 1.19 cells after 1 and 24 h incubation with the bioceramics in concentrations of 1 mg/mL, 5 mg/mL, and 10 mg/mL, the H_2_DCFDA fluorescent probe (Thermo Fisher Scientific) was used. H_2_DCFDA, after deacetylation to H_2_DCF (2′,7′-dichlorodihydrofluorescein), is oxidized intracellularly to highly fluorescence-emitting 2′,7′-dichlorofluorescein (DCF)^30^. Fluorescence was measured at the excitation wavelength of 495 nm and an emission wavelength of 525 nm using a SpectraMax® i3x Multi-Mode Microplate Reader (Molecular Devices, San Jose, CA, USA).

### Quantification of osteoconductive markers

To assess the osteoconductive potential, hFOB 1.19 osteoblasts were grown in the milieu of a particular nanoHAP under osteoinductive conditions (39 °C; differentiation medium: DMEM F-12 without phenol red, with 1% bovine serum, containing G148 geneticin and stimulants: β-glycerophosphate, ascorbic acid, dexamethasone, and the addition of the tested samples at a concentration of 5 mg/mL). As previously described, at the defined time points (7, 14, 21, and 28 days), the activity of alkaline phosphatase (ALP), cell proliferation, and the release of osteocalcin (OC) and interleukin-6 (IL-6) were quantified^31,32^. Briefly, cell proliferation was evaluated based on DNA quantification by CyQuant® assay (Thermo Fisher Scientific). The activity of ALP was assessed by a p-NPP (para-nitrophenyl phosphate) hydrolysis assay. The concentrations of OC and IL-6 in the culture media of hFOB 1.19 were determined by enzyme-linked immunosorbent assay (ELISA, R&D Systems, Minneapolis, MN, USA) according to the manufacturer's instructions. The sensitivity limit of the ELISA test for OC and IL-6 was 156.5 pg/mL and 9.38 pg/mL, respectively^32^.

### Statistical analysis

The statistical analysis was performed using GraphPad Prism 6 software (GraphPad Software, San Diego, CA, USA). Intergroup outcomes were compared for statistical significance using one-way or two-way ANOVA (analysis of variance). The differences were considered significant at the *p*-value < 0.05. The experiments were carried out in at least triplicate.

## Results

### Phase composition determination

**Figure 1:**
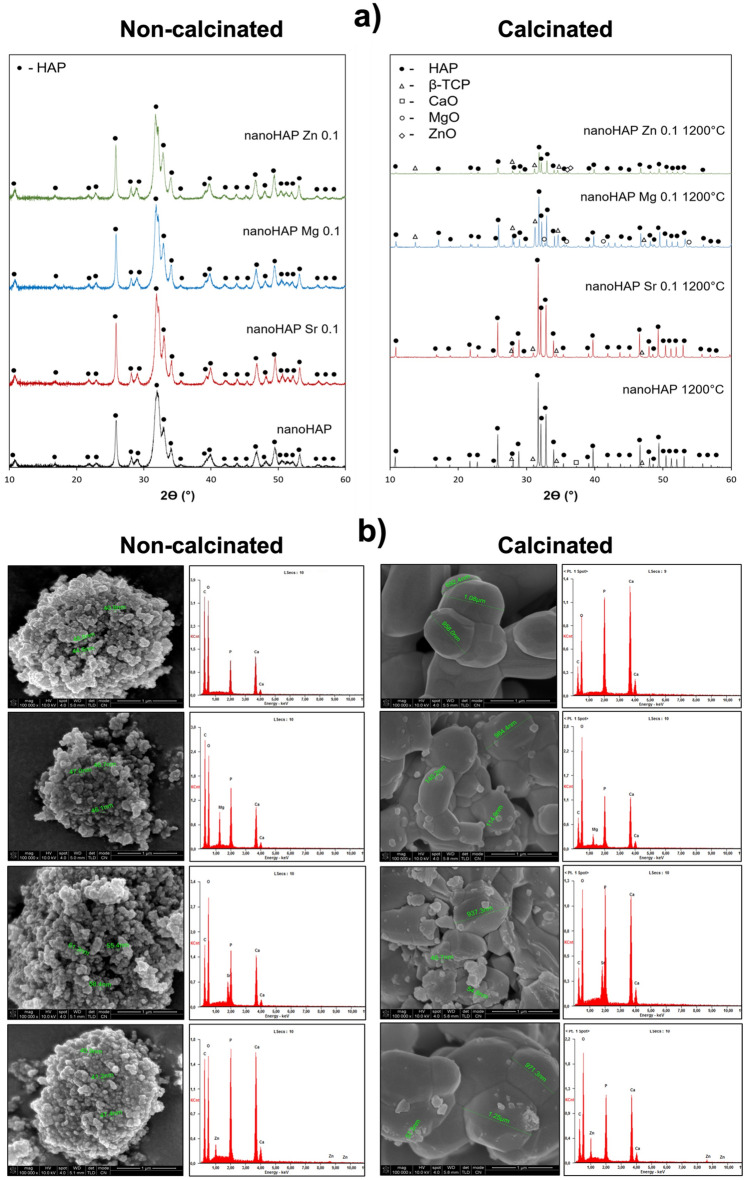
X-ray diffraction (**a**) and scanning electron microscope micrographs and EDS analysis (**b**) for the pure and ion-substituted hydroxyapatite samples before and after calcination at 1200 °C.

The X-ray diffraction patterns of the pure and Sr^2+^-, Mg^2+^-, and Zn^2+^-doped nanohydroxyapatite are shown in Fig. 1a. All samples revealed a similar pattern characteristic for the hydroxyapatite phase. The calculated lattice cell parameters of investigated powders are presented in Table 2. HAP doped with Sr^2+^ presented increased *a* and *c* parameters, thus an increase of the HAP cell volume (from 528.60 to 532.18 Å^3^). The inverse relationship was observed for nanoHAP Mg 0.1 where all lattice cell parameters decreased (V* = 527.99 Å^3^). In case of nanoHAP Zn 0.1, the *a* and *c* parameters increased and decreased, respectively, and cell volume remained practically unchanged (528.19 Å^3^). The small differences in the lattice cell parameters between unsubstituted and substituted HAP powders, as well as the absence of additional phases may suggest that all ions have been successfully incorporated into the hydroxyapatite structure. The broad peaks of low intensity confirm the low crystallinity and small grain size of the prepared nanohydroxyapatites. The calculated crystallite size was around 17–28 nm (Table 3). After calcination, the hydroxyapatite peaks became sharper due to increased crystallinity and crystal growth. Depending on the ion used, the size of the crystallites differed: 414 nm for nanoHAP control 1200 °C, 365 nm for nanoHAP Sr 0.1 1200 °C, 219 nm for nanoHAP Mg 0.1 1200 °C, and 244 nm for nanoHAP Zn 0.1 1200 °C. The calcination of HAP at 1200 °C revealed differences in the thermal stability of the synthesized powders (Fig. 1a, Table 3). It was shown that modifying nanoHAP with Sr^2+^, Mg^2+^, or Zn^2+^ contributes to its faster decomposition, which is the indirect confirmation of nanoHAP ionic modification^17,18^. The XRD patterns of thermally treated materials showed that the additional phase in the HAP structure is β-TCP. For nanoHAP, nanoHAP Mg 0.1, and nanoHAP Zn 0.1, diffraction peaks corresponding to CaO, MgO, and ZnO phases were identified, in addition to β-TCP. The highest proportion of β-TCP was observed in nanoHAP Mg 0.1 (44.88%). The calculated lattice cell parameters of HAP and β-TCP revealed differences between non-ion-substituted and substituted powders. The differences were observed in both phases for all samples. The nanoHAP Sr 0.1 1200 °C exhibited an increase in the HAP and β-TCP unit cell volume in comparison to a non-substituted powder (a and c increase). In the case of nanoHAP Mg 0.1 1200 °C and nanoHAP Zn 0.1 1200 °C lattice cell volume of HAP remained practically unchanged, whereas the cell volume of β-TCP was decreased. It is especially noticeable for HAP doped with Mg ions. The observed changes resulted from a minor expansion along the a-axis of the HAP lattice and a significant contraction along the c-axis for β-TCP. The data suggest that after calcination Sr ions were included in both HAP and β-TCP crystal structure, but Mg and Zn ions were present mainly in the β-TCP phase^33^.

**Table 2:** Lattice cell parameters of investigated powders.

Material	HAP			β-TCP		
	a = b (Å)	c (Å)	V* ( Å^3^)	a = b (Å)	c (Å)	V* ( Å^3^)
nanoHAP control	9.4136	6.8880	528.60	–	–	–
nanoHAP Sr 0.1	9.4390	6.8972	532.18	–	–	–
nanoHAP Mg 0.1	9.4116	6.8828	527.99	–	–	–
nanoHAP Zn 0.1	9.4183	6.8756	528.19	–	–	–
nanoHAP control 1200 °C	9.4143	6.8849	528.45	10.3898	37.2978	3486.81
nanoHAP Sr 0.1 1200 °C	9.4520	6.9209	535.48	10.4459	37.5262	3546.15
nanoHAP Mg 0.1 1200 °C	9.4195	6.8827	528.87	10.3647	37.1883	3459.76
nanoHAP Zn 0.1 1200 °C	9.4073	6.9070	529.37	10.3831	37.2919	3481.78

**Table 3:** The phase composition of the investigated nanohydroxyapatites before and after calcination at 1200 °C.

Non-calcinated				Calcinated (1200 °C)			
Material	Phase (COD)	Content (%)	Crystallite size (nm)	Material	Phase (COD)	Content (%)	Crystallite size (nm)
nanoHAP control	Hydroxyapatite (9001233) Ca_10_(OH)_2_(PO_4_)_6_	100	18	nanoHAP control 1200 °C	Hydroxyapatite (9001233) Ca_10_(OH)_2_(PO_4_)_6_	93.77	414
					Tricalcium phosphate (1517238) Ca_3_(PO_4_)_2_	5.69	72
					CaO (9006694)	0.54	146
nanoHAP Sr 0.1		100	28	nanoHAP Sr 0.1 1200 °C	Hydroxyapatite (9001233) Ca_10_(OH)_2_(PO_4_)_6_	88.64	365
					Tricalcium phosphate (1517238) Ca_3_(PO_4_)_2_	11.36	57
nanoHAP Mg 0.1		100	25	nanoHAP Mg 0.1 1200 °C	Hydroxyapatite (9,001,233) Ca_10_(OH)_2_(PO_4_)_6_	50.97	219
					Whitlockite (9012415) Ca_9_MgH(PO_4_)_7_	44.88	68
					MgO (4001314)	4.16	80
nanoHAP Zn 0.1		100	17	nanoHAP Zn 0.1 1200 °C	Hydroxyapatite (9001233) Ca_10_(OH)_2_(PO_4_)_6_	73.76	244
					Tricalcium phosphate (1517238) Ca_3_(PO_4_)_2_	25.61	77
					ZnO (2107059)	0.63	36

### Morphological characterization

The scanning electron microscope (SEM) indicated a globular morphology and agglomeration behavior, typical of precipitated nanoHAPs, and confirmed the nominal substitution of Mg^2+^ or Sr^2+^ or Zn^2+^ for both non-calcinated and calcinated nanoparticles. The chemical composition of the synthesized products was determined by the energy dispersive spectroscopy (EDS) analysis shown in Fig. 1b. The average particle size was determined from dozens of measurements from SEM images as follows: the size of non-calcinated sphere-like shape particles ranged 36.5–49.6 nm for pure nanoHAP, 40.8–48.1 nm for Mg^2+^, 53.6–61.3 nm for Sr^2+^, and 40.9–49.5 nm for Zn^2+^ doped nanoHAP, which confirms the nanometric size of the hydroxyapatite particles. The measured particle sizes were significantly bigger than those calculated from the XRD pattern, which confirms the tendency of particles to agglomerate. The sizes of calcinated particles ranged form 108.0–1.08 µm for nanoHAP; 114.9–984.4 nm for Mg^2+^, 46.7–937.3 nm for Sr ^2+^, and 39.1–971.3 nm for Zn^2+^ doped nanoHAP. The SEM micrographs (Fig. 1b) confirm that calcination affected the morphology of hydroxyapatites and resulted in the diffusion of the particles to form a bigger irregular structure with a nearly round shape and interconnected fine particles and pores.

### Specific surface area (BET)

Supplementary Fig. S1 online presents the BET isotherms and BET surface area plots for materials before and after calcination. The calculated BET-specific surface areas are presented in Table 4. The surface area of the nanoHAP modified with Zn^2+^ was greater (100.1 m^2^/g) than nanoHAP modified with Mg^2+^ (76.4 m^2^/g) or Sr^2+^ (66.1 m^2^/g). However, after the calcination process, the surface area dropped considerably in the obtained samples, with the highest value reached for the material with magnesium presence. The obtained specific surface areas were compatible with calculated crystallite size of powders (Table 3) and increased with decreasing the crystallite size of the materials studied.

**Table 4:** Specific surface area of powder before and after calcination.

Material	Specific surface area [m^2^/g]	
	Non-calcinated	Calcinated at 1200 °C
nanoHAP	80.7	0.3310
nanoHAP Sr 0.1	66.1	0.3705
nanoHAP Zn 0.1	100.1	0.6005
nanoHAP Mg 0.1	76.4	1.3333

### Fourier—transform infrared spectroscopy (FTIR)

**Figure 2:**
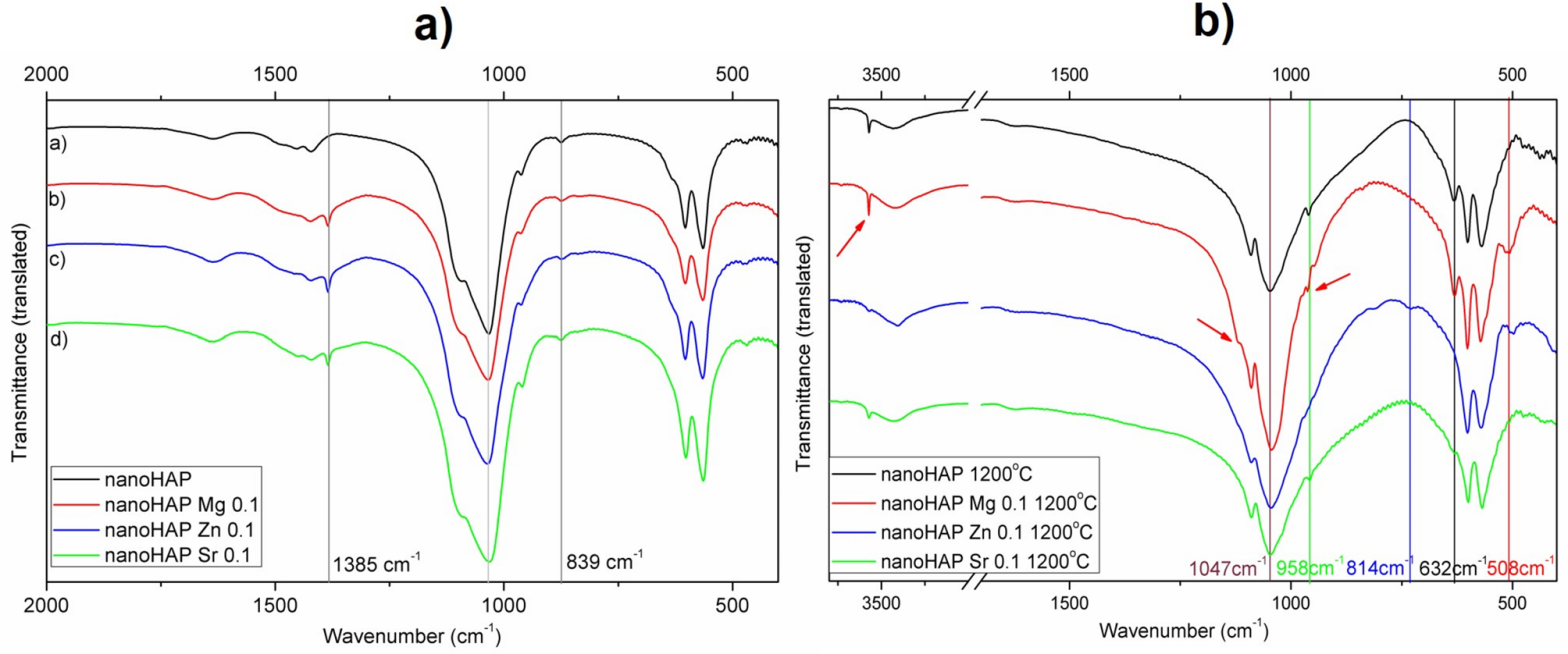
Infrared spectra of materials (**a**) before calcination, (**b**) after calcination at 1200 °C.

Figure 2a shows the FTIR spectra of nanohydroxyapatite before calcination. Almost all characteristic bands for hydroxyapatite can be identified^34^. The medium intense band at about 3572/cm is due to the vibrations of hydroxyl groups on the HAP surface. The bands observed in the range of 3237–3422/cm and at 1634/cm are related to the presence of water. Medium bands at about 1453/cm, 1421/cm, and 874/cm are assigned to ν_3_ C–O (CO_3_^2−^) vibrations. The four vibrational modes associated with phosphate ions were identified at ν_1_ 962/cm, ν_2_ 468/cm, ν_3_ 1092 + 1033/cm, ν_4_ 603 + 562/cm, while the band at 636/cm can be assigned both to the O–H and labile PO_4_^3−^^35^. The absence of a band at about 1190/cm may result from different phosphate chemical environments.

The Mg, Sr, and Zn nitrate precursors partially replaced calcium ions in the apatite structure. In the spectra of modified hydroxyapatite before calcination, additional peaks were observed at 1385/cm and 839/cm, which is related to the presence of unreacted nitrates (Fig. 2a). Therefore, the signals of phosphates are partially masked and can be distinguished only after thermal treatment and nitrate degradation (temperature above 500 °C). In nanoHAPs doped with magnesium, a weak band at 838/cm may be related to MgO presence. Other characteristic bands for MgO were not observed or overlapped with similar bands, i.e., the signals at 1385/cm and 3412/cm.

To follow the structural changes during calcination, FTIR spectra of the material after thermal treatment were compared and are presented in Fig. 2b. After calcination at 1200 °C some specific bands disappear, for example, those most prominent for nitrates at 1385/839/cm, and carbonates at 1453/1421/cm and 875/cm. This is consistent, as thermal degradation of stable ions occurs at temperatures above 500 °C. Moreover, some bands shift by ± 4/cm; for example, the band at 1033/cm and the weak band around 1382/cm are shifted. These effects are probably related to the differences between nanoHAP materials regarding hydroxyl and phosphate bands which may suggest a different substitution of HAP sites as well as the formation of different apatites. Calcination leads to the condensation of surface hydroxyl groups with the formation of water, which was proved by the presence of bands at 3734/cm followed by the ones above 3400/cm.

### Thermal behavior

**Figure 3:**
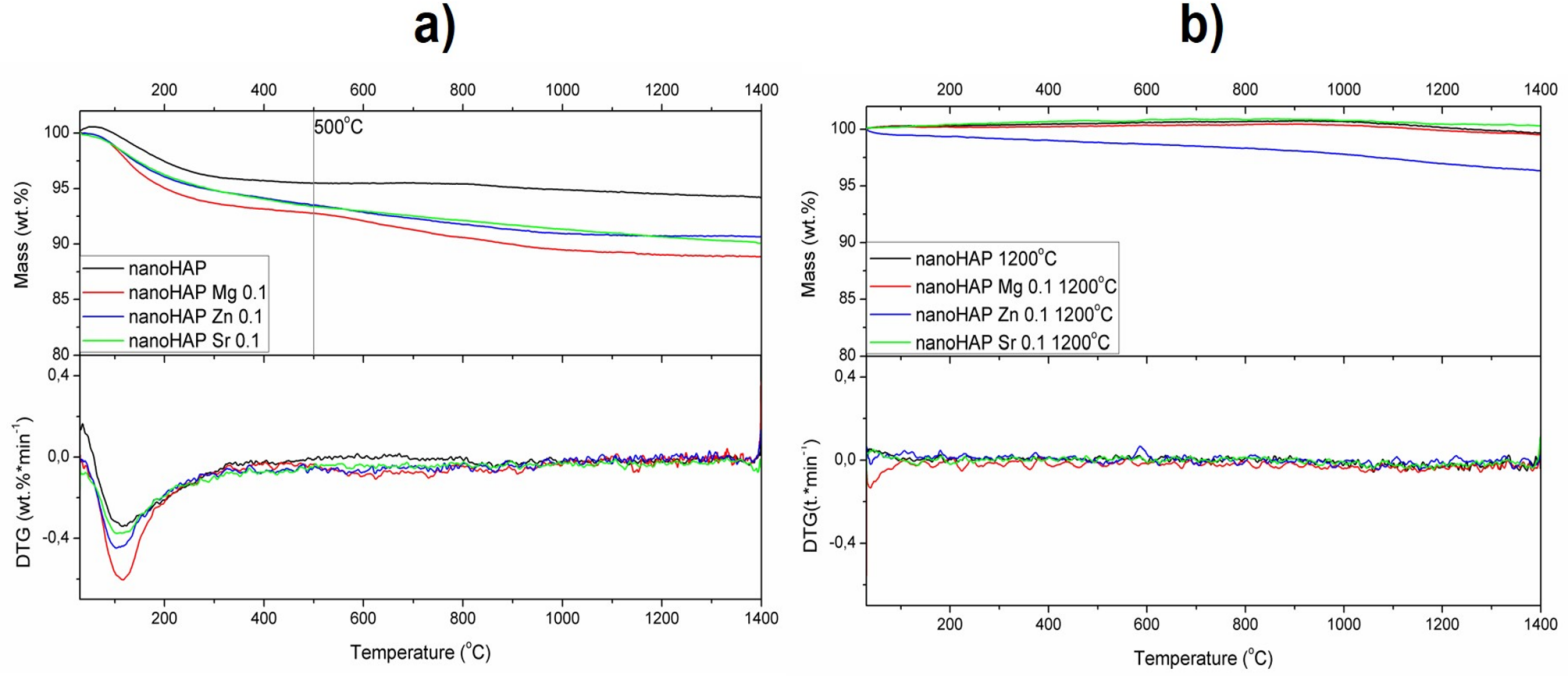
Thermal analysis curves for nanoHAP and modified nanoHAP before (**a**) and after calcination (**b**), assessed by thermogravimetry.

The thermal behavior of nanoHAP was investigated during TG–DTA experiments in the inert atmosphere of argon. Thermogravimetric (TG) curve of the unmodified nanoHAP showed an initial mass loss of about 4 wt.% related to the evaporation of physically adsorbed water on the surface of the particles and chemically adsorbed water, as well as crystal and lattice water loss up to 400 °C. It is impossible to distinguish mass losses in this region, most probably because the measurements were conducted with lids on the top of the Al_2_O_3_ crucible. Despite the lid has a small hole to ensure the gas flow, some limitations related to the gas diffusion into the sample are possible. Simultaneously, the use of lids prevents the nanoparticles’ escape from the crucible. TG curves for the modified ceramics show higher mass loss than those for nanoHAP, indicating the decomposition of residual reagents (nitrates) and possibly the constituents of the nanoHAP (carbonates, hydroxyls). For HAP, dehydroxylation of surface phosphorus occurs in the temperature range of 450–730 °C, whereas dehydroxylation of hydroxyapatite can take place at higher temperatures of 730–1020 °C^36^. It has been reported that dehydroxylation of HAP starts at about 800 °C and leads to the decomposition of HAP to oxyapatite Ca_10_(PO_4_)_6_O (Fig. 3)^37,38^.

### Analysis of the ion release profile

**Figure 4:**
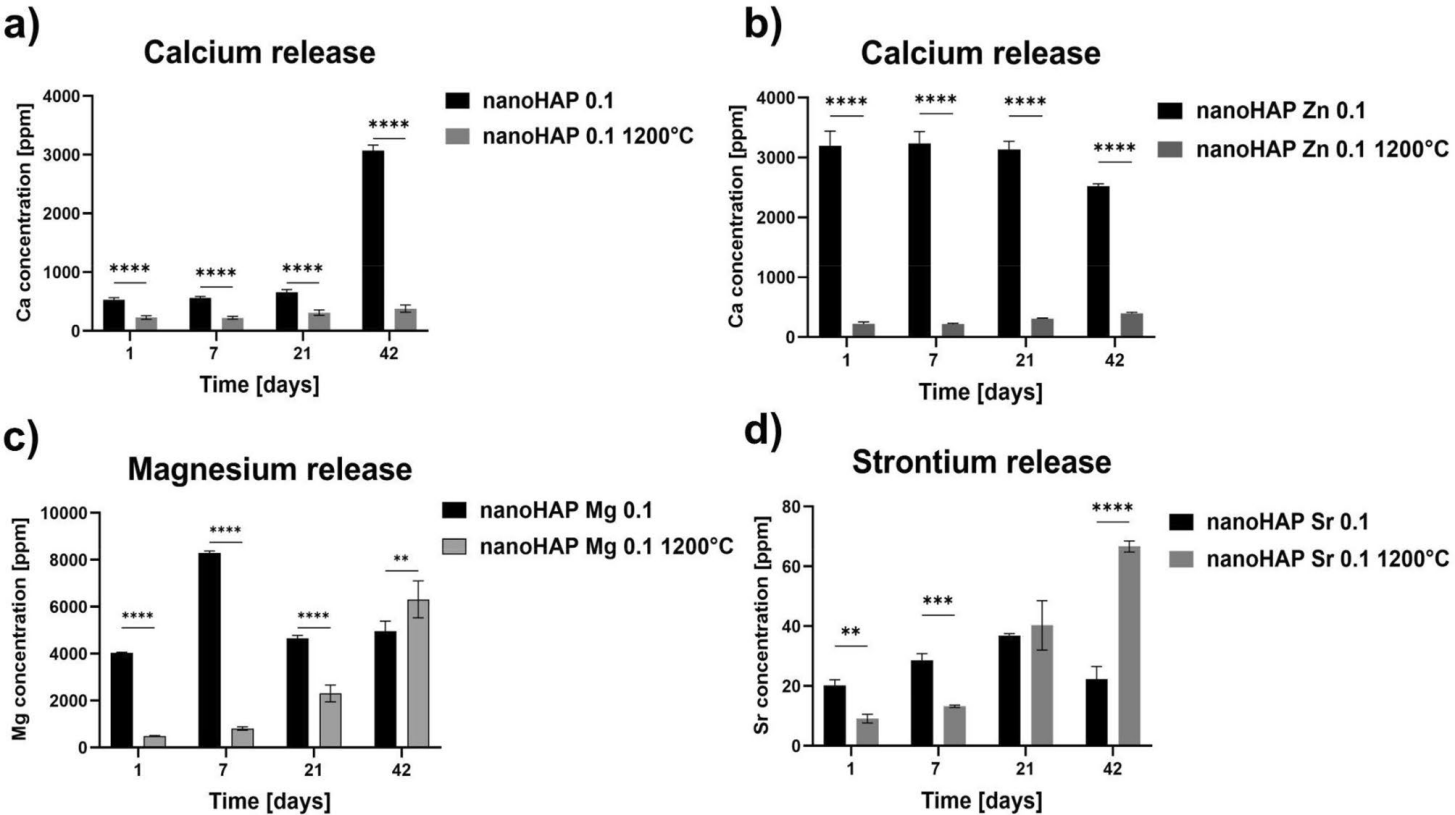
Release profiles of calcium ions from calcinated and non-calcinated samples of nanoHAP (**a**) and nanoHAP Zn 0.1 (**b**), of magnesium ions**,** from nanoHAP Mg 0.1 (**c**), and strontium ions from nanoHAP Sr 0.1 (**d**); for calcinated and non-calcinated samples. The release was measured by inductively-coupled plasma—optical emission spectroscopy. Data are presented as mean values and standard deviation of assay triplicates. *p < 0.001 between non-calcinated and calcinated samples based on the two-way ANOVA (Tukey’s) evaluation results.

Figure 4 shows the release profile of Ca^2+^ for nanoHAP and nanoHAP Zn 0.1 and the profile of Mg^2+^ and Sr^2+^ ions released from nanoHAP Sr 0.1, nanoHAP Mg 0.1, both in non-calcinated and calcinated samples. The test was performed in static conditions (without changing the medium) in deionized water at 37 °C for 1, 7, 21, and 42 days. Unfortunately, zinc ion release levels were under the detection limit of ICP-OES and could not be monitored. That is why for zinc-modified HAP, only calcium release was determined.

The results demonstrated that if the nanoHAP was doped with zinc ions, the level of calcium release increased almost six times (Fig. 4a, b). For unmodified HAP, an interesting fact of a significant increase of calcium release after 42 days of incubation was observed, which needs to be further clarified. The calcination process led to a meaningful decrease in the calcium release level, probably due to the stabilization of ions in the hydroxyapatite structure and reduction of the surface area of the tested powders.

Non-calcinated nanoHAP Mg 0.1 showed a burst release of Mg^2+^ ions from the 1st until the 7th day of incubation, ranging from 4000 to 8000 ppm. After 7 days, the release of Mg^2+^ was decreased to 4200 ppm. Long-term magnesium release was maintained at a similar level until the end of the experiment (Fig. 4c). In this case, calcination, also leads to a significant decrease in magnesium release. However, after 21 days of incubation, an increase was observed again. Regarding controlled ion supplementation, this is a very promising phenomenon.

For HAP modified with Sr^2+^ (Fig. 4d), significantly lower values of ion release were observed, and this level increases slightly until 21 days of incubation, while after this time decreases to the starting level. Analysis of the calcinated HAP substituted with Sr^2+^ showed that ion release raised until 42nd day of incubation, and the highest value jump was registered after 21 days of incubation.

## Biological properties

### In vitro cytocompatibility

**Figure 5:**
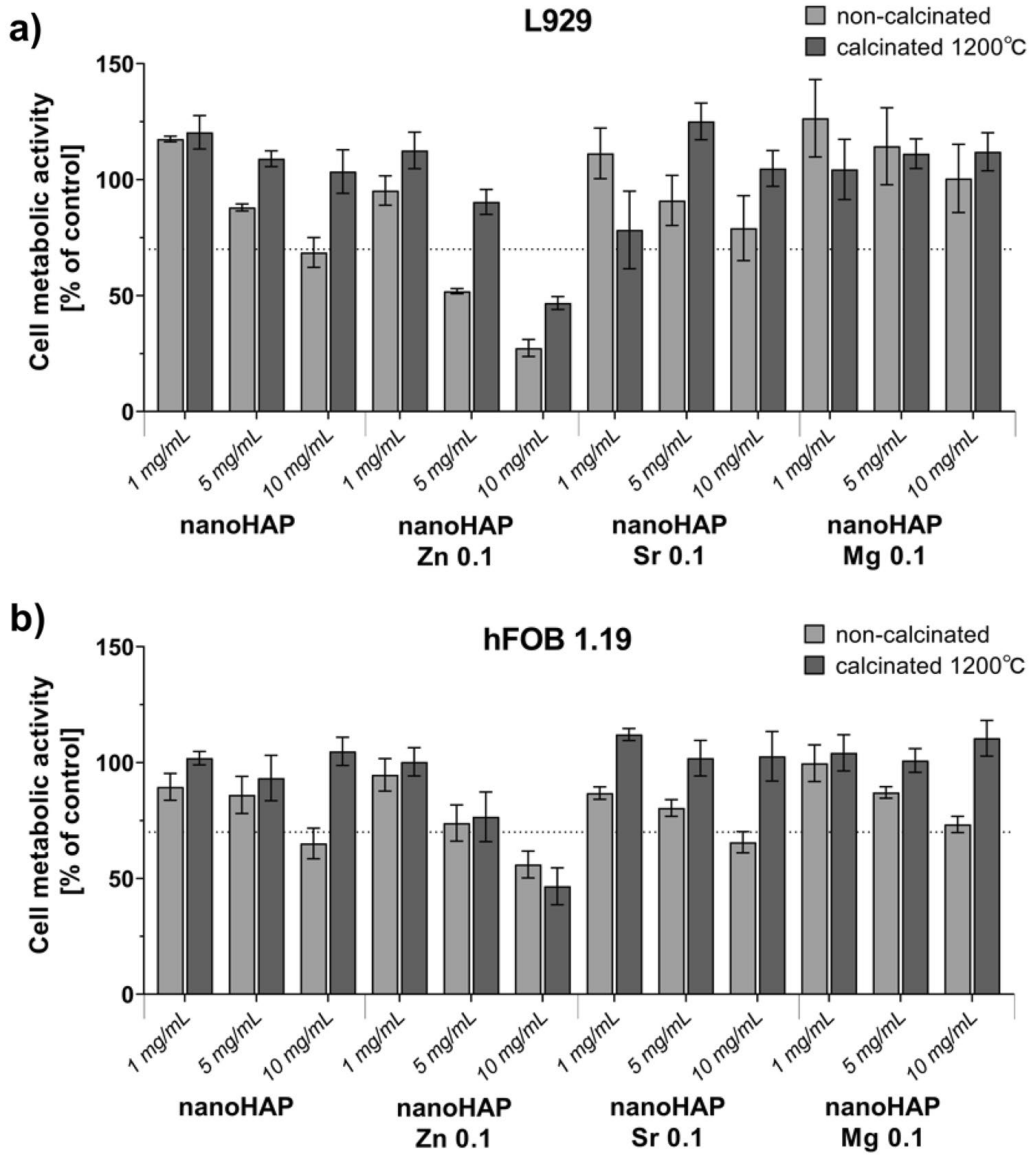
The MTT reduction assay was used to assess the viability of fibroblasts (**a**) and osteoblasts (**b**) after 24 h of incubation with non-calcinated or calcinated (1200 °C) nanoHAP. Data are presented as mean viability calculated compared to control (cells in medium) and standard deviation of assay triplicates. L929: K1: 100.0% ± 9.4%; K2: 20.2% ± 2.1%. hFOB 1.19: K1: 100.0% ± 7.3%; K2: 4.1% ± 0.1%. K1—viability control (cells in culture medium without the test sample), and K2—cytotoxicity control, where cells were treated with 2% saponin, a highly cytotoxic substance.

The effect of non-calcinated and calcinated (1200 °C) nanoHAP materials on the metabolic activity of L929 fibroblasts (Fig. 5a) and human hFOB 1.19 osteoblasts (Fig. 5b), was investigated. The prepared materials were tested in several concentrations, 1, 5 and 10 mg/mL. The viability of mouse fibroblasts and human osteoblasts incubated for 24 h in the presence of non-calcinated nanoHAP was dependent on the concentration of nanoHAP remaining in contact with cells. The cell viability decreased in the milieu of higher doses of this material. Moreover, calcination of the samples at 1200 °C caused a notable increase in the cell viability (L929 and hFOB), and this effect was not dose-dependent, except for nanoHAP Zn 0.1. The non-calcinated samples of nanoHAP (1 and 5 mg/mL), nanoHAP Zn 0.1 (1 mg/mL), nanoHAP Sr 0.1 (1, 5 and 10 mg/mL), nanoHAP Mg 0.1 (1, 5 and 10 mg/mL) and calcinated samples of nanoHAP 1200 ℃ (1, 5 and 10 mg/mL), nanoHAP Zn 0.1 1200 ℃ (1 and 5 mg/mL), nanoHAP Sr 0.1 1200 ℃ (1, 5 and 10 mg/mL), nanoHAP Mg 0.1 1200 ℃ (1, 5 and 10 mg/mL) did not cause a decrease in L929 cells’ metabolic activity below 70%. Thus, these materials were confirmed to be cytocompatible on in vitro levels.

### ROS production

**Figure 6:**
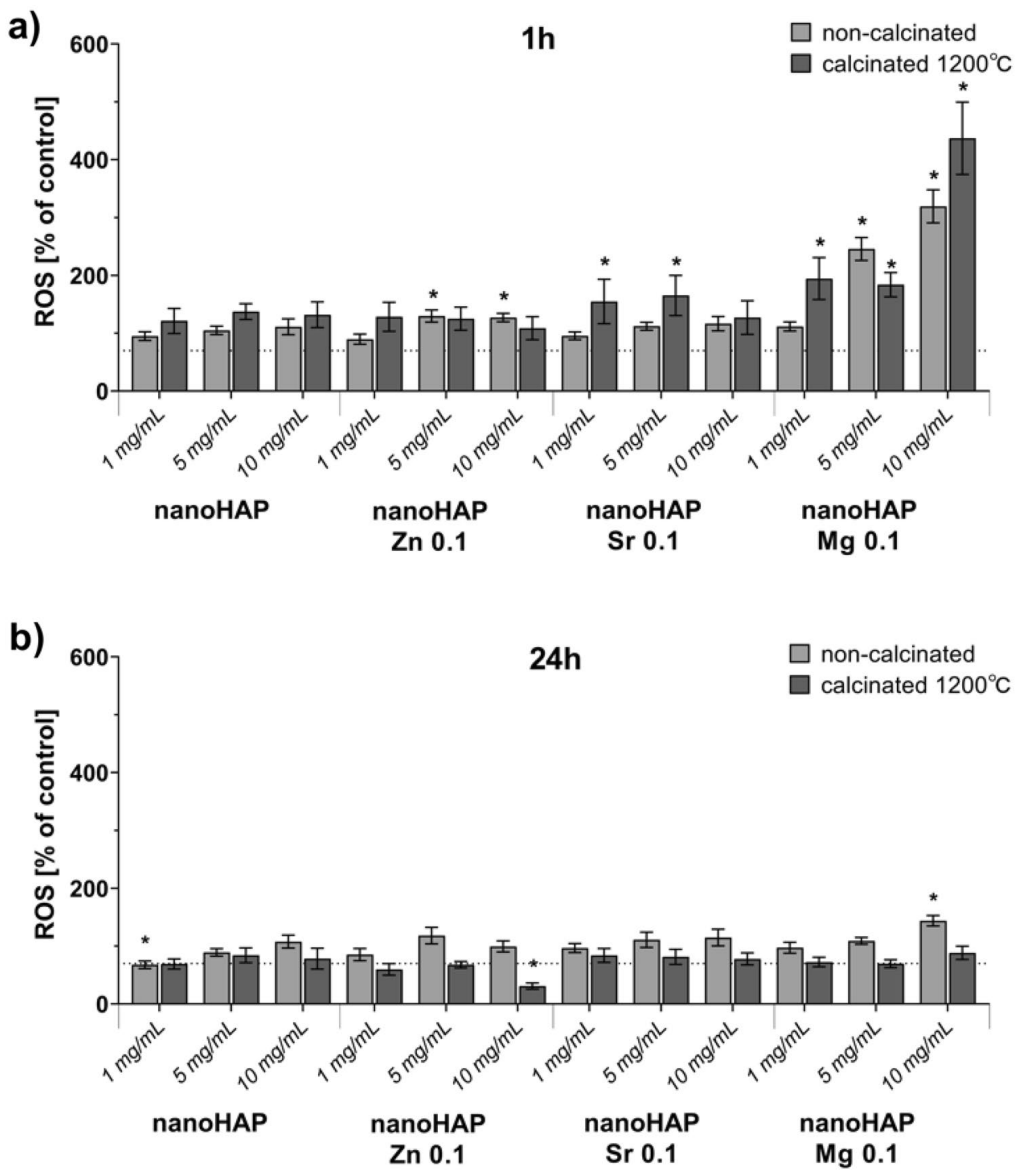
Generation of reactive oxygen species (ROS) in hFOB 1.19 cells after exposure to non-calcinated and calcinated (1200 °C) nanoHAP and nanoHAP modified with Zn, Sr, and Mg after 1 h (**a**) or 24 h (**b**) incubation measured using an H_2_DCFDA probe. Data are presented as mean values and standard deviation of assay triplicates. *p < 0.001 between non-calcinated and calcinated samples vs. control, based on the two-way ANOVA (Tukey’s) evaluation results.

In order to evaluate how nanoHAP affects the production of reactive oxygen species (ROS) in human osteoblasts, an H_2_DCFDA probe was utilized. The results presented in Fig. 6a, b show that 1 h incubation of hFOB 1.19 cells in the presence of nanoHAP Mg 0.1, both raw and calcinated at 1200 °C, significantly increased ROS levels. However, after 24 h, the increase in the ROS level was the highest only after incubation with non-calcinated nanoHAP Mg 0.1 in the concentration of 10 mg/mL. A significant increase of over 150% in ROS production was observed for calcinated nanoHAPs modified with strontium (1 and 5 mg/mL) after 1 h of incubation. The observed effect was not dose-dependent. For nanoHAP 1 mg/mL and nanoHAP Zn 0.1 10 mg/mL, a statistically significant decrease in the ROS levels after 24 h of incubation was noticed.

### Osteoconductive potential

**Figure 7:**
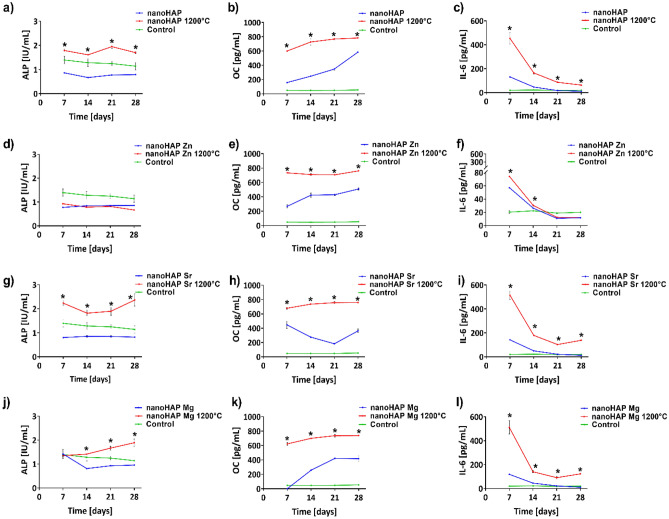
NanoHAP-driven production of ALP (**a**, **d**, **g**, **j**), OC (**b**, **e**, **h**, **k**), and IL-6 (**c**, **f**, **i**, **l**) in hFOB1.19 cell cultures exposed to non-calcinated or calcinated nanoHAP or modified with Zn, Sr, and Mg after 7, 14, 21 and 28 days. The results represent the mean values ± SEM. *p < 0.05 between the calcinated and non-calcinated samples, based on the one-way ANOVA (Kruskal–Wallis test) evaluation results.

The osteoconductive potential of nanohydroxyapatites was analyzed and presented in Fig. 7. The results indicate that the calcination of the samples at 1200 °C led to a significant increase in ALP secretion by hFOB 1.19 compared to the concentration of this osteoblastic marker detected in cell cultures incubated with the non-calcinated samples (Fig. 7a, g, j). The amounts of ALP detected in 28-day osteoblast cultures incubated with calcinated nanoHAP and ion-modified nanoHAP reached: 1.7 ± 0.02 IU/mL (nanoHAP), 2.4 ± 0.5 IU/mL (nanoHAP Sr 0.1), 1.9 ± 0.3 IU/mL (nanoHAP Mg 0.1), and were significantly higher (p < 0.05) as compared to ALP level detected in the presence of non-calcinated specimens: 0.8 ± 0.02 IU/mL (nanoHAP), 0.8 ± 0.05 IU/mL (nanoHAP Sr 0.1), 1.0 ± 0.01 IU/mL (nanoHAP Mg 0.1). Interestingly, no differences between the levels of ALP produced by osteoblasts cultured with non-calcinated nor calcinated nanoHAPs modified with Zn (Fig. 7d) were observed. Moreover, ALP production was lower than the activity detected in the control cell cultures at all time points. The production of osteocalcin, another key marker of osteoblast differentiation, was significantly higher in hFOB 1.19 cell cultures incubated with calcinated nanoHAPs at all time points, compared to the level of OC detected in cell cultures exposed to non-calcinated samples, 599.3 ± 21.6 pg/mL and 157.8 ± 8.9 pg/mL, p < 0.0001 (on day 7) and 783.3 ± 0.9 pg/mL and 584.0 ± 9.3 pg/mL, p < 0.0001 (on day 28), respectively (Fig. 7b). Significant differences were also noticed between the calcined and non-calcined nanoHAPs with Zn, Sr, and Mg, which confirmed the beneficial effect of calcination at 1200 °C and ionic modification on increased OC production in hFOB 1.19 cells (Fig. 7e, f, k).

The production of IL-6 decreased throughout the entire course of the experiment. However, at all time points, the level of IL-6 produced by hFOB 1.19 cultured on calcinated nanoHAPs was higher (63.0 ± 5.3 pg/mL) than that detected in cultures exposed to non-calcinated samples (11.6 ± 0.2 pg/mL), p = 0.03 (Fig. 7c). This trend in IL-6 secretion was also demonstrated for calcinated vs. non-calcinated nanoHAPs modified with Zn, Sr, and Mg (Fig. 7c, f, i, l).

**Figure 8:**
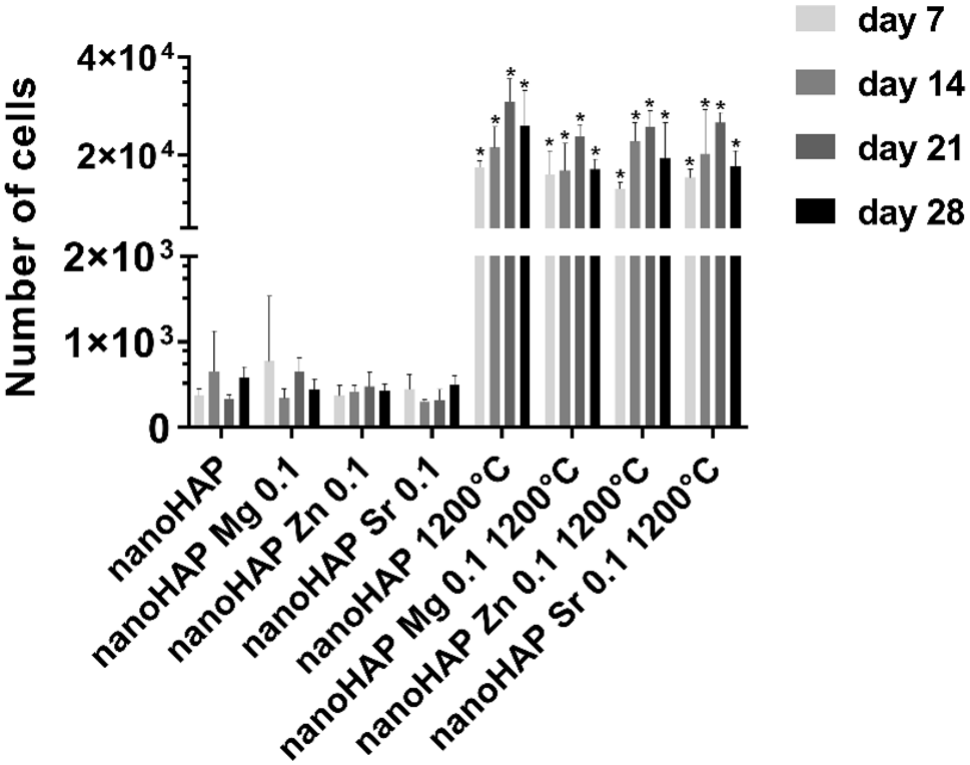
Proliferation of hFOB 1.19 osteoblasts after 7, 14, 21, and 28 days of incubation with the non-calcinated and calcinated (at 1200 °C) nanoHAP and nanoHAP modified with Zn, Sr, and Mg. The results are expressed as mean relative DNA content (RDC) ± SD calculated from three independent experiments. *p < 0.05 between non-calcinated and calcinated samples, based on the two-way ANOVA (Tukey’s) evaluation results.

DNA quantification was performed at different time points to assess the influence of fabricated nanoHAP materials and their modification on the long-term viability of osteoblast cultures. In this assay, the fluorescence intensity of DNA corresponds to the number of cells and thus the measurement of cell proliferation is possible. The results indicated that calcination of the samples at 1200 °C promoted efficient osteoblast proliferation (Fig. 8). The mean number of cells (MNoC) present within calcinated nanoHAPs after 28 days were significantly higher (p < 0.05) than in non-calcinated specimens: nanoHAP (5.9 × 10^2^ ± 1.2 × 10^2^ vs. 2.6 × 10^4^ ± 7.2 × 10), nanoHAP Zn 0.1 (4.3 × 10^2^ ± 8.3 × 10^2^ vs. 1.9 × 10^4^ ± 1.1 × 10^3^), nanoHAP Sr 0.1 (5.0 × 10^2^ ± 1.1 × 10^2^ vs. 1.8 × 10^4^ ± 3.2 × 10^3^), and nanoHAP Mg 0.1 (4.4 × 10^2^ ± 1.3 × 10^2^ vs. 1.7 × 10^4^ ± 2.1 × 10^3^).

## Discussion

Currently, nanohydroxyapatite is widely used in bone regeneration and it is crucial to investigate its possible further improvements. Therefore, this study aimed to understand the influence of ion doping and calcination on HAP properties and biological activity. The paper presents characterization of nanohydroxyapatite synthesized by precipitation from an aqueous solution, and further modified with Mg^2+^, Sr^2+^, and Zn^2+^. Moreover, the effect of calcination at 1200 °C was correlated with the influence of the ion modification.

A thorough characterization of the chemical structure, phase composition, and microstructure of the nanoHAP and its modifications was performed. The FTIR and XRD analyses confirmed the presence of hydroxyapatite as an only phase. The absence of additional phases in modified HAP may suggest that all ions have been successfully incorporated into the hydroxyapatite structure. These results are in agreement with other data, which showed a preferential substitution of cations in the Ca (I) and Ca(II) position^39,40^. The XRD analysis showed that incorporating Sr^2+^, Mg^2+^, and Zn^2+^ into nanoHAP resulted in a structural modification of the HAP lattice due to differences in the size of individual ions, with the following ionic radiuses:

$${\text{Mg }}\left( {0.{72}\;{\text{\AA}}} \right){\text{ < Zn }}\left( {0.{74 }\;{\text{\AA}}} \right){\text{ < Ca }}\left( {0.{99 }\;{\text{\AA}}} \right){\text{ < Sr }}\left( {1.{12}\;{\text{\AA}}} \right)$$

The differences in ionic radius between Ca^2+^ and other ions leads to strong distortions of the HAP lattice and reduces its crystallinity. It has a direct impact on increasing the solubility and biodegradability of substitute HAP compared to unmodified analogues and favors their thermal conversion into substituted β-TCP^40^, which was also confirmed here.

The observed calcination process led to significant structural changes, as proved by FTIR (disappearance of particular bands) and XRD (formation of additional phases). After calcination, the quantity of HAP decreases, and β-TCP is formed as the second most prominent phase. The highest proportion of β-TCP was observed in nanoHAP Mg 0.1 (44.88%), then in nanoHAP Zn 0.1 (25.61%), nanoHAP Sr 0.1 (11.36%), and finally the smallest in pure nanoHAP (5.69%). In calcinated nanoHAP, nanoHAP Mg 0.1, and nanoHAP Zn 0.1, diffraction peaks corresponding to CaO, MgO, and ZnO phases were identified, in addition to β-TCP. This is in line with previous reports, which showed that at higher temperatures (above 1000 °C), hydroxyapatite could convert to TCP and calcium oxide (CaO) phases following the equation below^39,41^:

$${\text{Ca}}_{{{\text{1}}0}} \left( {{\text{PO}}_{{\text{4}}} } \right)_{{\text{6}}} \left( {{\text{OH}}} \right)_{{\text{2}}} \to {\text{ 3Ca}}_{{\text{3}}} \left( {{\text{PO}}_{{\text{4}}} } \right)_{{\text{2}}} + {\text{ CaO }} + {\text{ H}}_{{\text{2}}} {\text{O}}$$

The coexistence of HAP with β-TCP and CaO strongly depends on the change in Ca/P molar ratio relative to the stoichiometric value of 1.667; even a slight variation in Ca/P ratio may result in significantly different phase composition^42^. A high proportion of β-TCP in the structure, related to the different 1.667 Ca/P ratio, can worsen the biological properties of bioceramics. On the other hand, the presence of CaO and a higher Ca/P ratio improve biological activity^41,43^. Moreover, it was shown that ionic substitution destabilizes the hydroxyapatite structure and favors its thermal conversion into β-tricalcium phosphate even at a lower temperature^44–50^. Furthermore, previous reports showed that as the calcination temperature increases, more of the β-TCP phase is formed, which is in line with our results^51^. The comparison of unit cell parameters of HAP and β-TCP in the non-substituted and substituted powders suggest that after the calcination process, the Sr ions were included in both HAP and β-TCP crystal structure, while Mg and Zn ions were mainly in the β-TCP phase.

No noteworthy observation comes from the thermal analysis of the materials, what may suggest that undetectable amounts of modifiers are present in the structure of nanopowders. The nano-sized material is tough to analyze in TG–DTA experiments, where the dynamic gas flow can cause the nanoparticles’ entrainment by the carrier gas. In addition, the bulk material inside the crucible can create an air cushion and interfere with the data recording. The only visible effect is that some hydroxy groups escape from the structure after calcination (mostly loosely bound water molecules).

In order to assess the impact of modifications on the chemical composition of HAPs, the morphology of the materials was analyzed using SEM equipped with an EDS detector. The calcination at 1200 °C led to the diffusion of particles, resulting in the creation of larger, more irregular, almost round shape structures. These structures displayed interconnected fine particles and pores. Similar results were presented by Aina et al.^16,52^, Mocanu et al.^17^, and Ofudje et al.^53^. Those studies reported the growth of various round-shaped crystals after calcination and substitution of magnesium, strontium, or zinc ions within the apatite structure. The presence of these particles, characterized by diverse sizes and an irregular, finely-textured morphology within the nanoHAP may increase their bioactivity. Ionic substitution in nanoHAP, involving both magnesium and strontium ions, influenced the morphology of HA. This observation finds support in the studies by Geng et al.^21,24^.

The results of the ion release test for the nanopowders showed that both ion modification and thermal treatment strongly affected the nanopowders' behavior. Following thermal treatment at 1200 °C, pure nanoHAPs changed their original performance, and ion release dropped significantly in all variants. This might have depended on several factors. Above all, a significant reduction in the specific surface area of nanoHAP grains after calcination may have mattered. The calcination-induced decrease in the size of the material's specific surface area capable of ion exchange seemed critical in reducing the ions’ release.

The most prominent release of ions was observed for calcinated magnesium-doped nanoHAP, which led to a gradually increasing Mg^2+^ release over time. Apart from the size of the specific surface area, the Mg^2+^ release may be related to the presence two phases of calcium phosphate: HAP and β-TCP after calcination, which was the largest in Mg-doped nanoHAPs. According to the literature, β-TCP has been recognized as a resorbable material^54,55^, while HAP is considered non-resorbable^56^. Hence, it can be inferred that the rate of ion release might be influenced by the degradation and resorption capabilities of the resulting material and the more β-TCP, the more ions are released. Furthermore, the XRD data detected the presence of magnesium oxide, which is known for its rapidly and fast release form materials. This finding adds another layer of explanation to the observed ion release pattern showed in the results here. Non-calcinated Mg-doped nanoHAP showed an initial burst release of Mg^2+^ ions, followed by a noticeable decline. These data are consistent with those presented by Khoshzaban et al.^57^ and Sprio^58^, this phenomenon could be related to the release of loosely bonded ions, resulting in faster ion loss rates at the initial stages of incubation^17,18^.

While analyzing ion release profiles from strontium- and magnesium-modified nanoHAPs, the release rates of Sr^2+^ were found to be significantly slower than those of Mg^2+^. This may be due to much stronger Sr^2+^ than Mg^2+^ bonding to the nanoHAP structure^59^.

The material most distinct from the others was zinc doped nanoHAP. The amount of zinc ions released was under the instrument detection limit, and only calcium ions’ release could be followed. A similar situation was observed by Sprio et al.^59^ and Mocanu A.^17^, even if the experiment was conducted for 90 days. In the case of the Zn-modified HAP, calcium release was observed. Calcium release from the calcined Zinc-modified nanoHAP exhibited relative stability, while surprisingly, in the non-calcinated Zn-modified HAP the calcium release level was significantly higher. Unfortunately, zinc ion release levels were under the detection limit. This might relate to the presence of PO_4_^3-^ groups in a solution which can affect the dissolution of Zn^2+^, as suggested by Li^60^. Absorbtion of zinc ions onto the surface may also play a role. Considering the behavior of Zn-modified HAP, it can be speculated that Zn^2+^ ions are located in energetically stable substitutional positions of the HAP and β-TCP phases^60^. Furthermore, the analysis of material specific surface area (SSA) revealed alterations in crystallite size, dependent on the incorporated ions, which consequently influenced the SSA of the synthesized powders. The existing literature highlights that optimal biomaterials for bone regeneration should exhibit such as a high specific surface area^61^. As demonstrated in this study, changes in crystallite size were accompanied by an increase in SSA, particularly in the non-calcined materials, promoting ion release and bioactivity. The nanoHAP calcined at 1200 °C exhibited a reduced specific surface area, which contributed to decline biodegradation. This might potentially account for observed to limited in the release of Mg^2+^, Zn^2+^, and Sr^2+^ ions after calcination. These results are consistent with those of Trzaskowska et al.^61^. Interestingly, calcination did not result in the loss of bioactivity and osteoconduction, making these materials attractive for bone regeneration.

To study regenerative potential of the investigated HAPs, biological characteristics was examined. Mg-, Sr-, and Zn-doped and calcinated nanoHAP, better promoted cell growth and proliferation than non-calcinated materials. The positive effect of ion-doped nanoHAP on osteoblast response was associated with improved cytocompatibility and cell activity, implying that nanoHAP doping ions affect metabolism during bone remodeling, in accordance to the results of Rapuntean et al.^62^. Both early and late markers of osteogenesis (osteocalcin and ALP, respectively) demonstrated that calcination and ion modification promoted high cell activity in osteoblast cell culture. This is in accordance with Mocanu et al.^17^, Rapuntean et al.^62^, de Lima et al.^63^ and Gnaneshwar et al.^64^, who reported that nanoHAP substituted with strontium, magnesium and zinc could enhance osteoconduction and osteoblast proliferation. Furthermore, calcinated materials were found to induce IL-6, a proinflammatory cytokine secretion which then gradually decreased. In the early stage, during fracture healing IL-6 supports regeneration and contributes to bone remodeling. However, excessive IL-6 production may lead to a chronic pathological inflammation, underscoring the need for careful assessment^36,65^. The production of IL-6 decreased throughout the entire duration of the experiment. Interestingly, calcinated materials induced a higher level of IL-6 compared to non-calcinated materials. Despite the presence of IL-6 production in the initial stages, its release did not impact the production of osteocalcin and alkaline phosphatase by osteoblastic cells. This is in line with other available data^17,66,67^.

Another parameter to assess biological effects was ROS production. ROS levels typically increase during the early stages of tissue regeneration, triggering a localized inflammatory response crucial for wound healing^68^. As demonstrated in this study, magnesium- and strontium-doped HAP, calcinated induced higher levels of ROS after 1 h incubation and decreased after 24 h. This pattern is considered to provide additional stimuli for cell regeneration^68^. Thus, calcined nanoHAP modified with magnesium and strontium exhibits such potential.

To summarize, this paper presents a detailed study on the fabrication and characterization of nanohydroxyapatite for biomedical applications. A simple method of producing nanoHAPs based on precipitation from aqueous solutions and involving dual modification, has resulted in bioceramics with the desired physicochemical and biological properties.

## Conclusions

Combining ion modification and calcination at 1200 °C demonstrates notable benefits for enhancing both the structural and biological attributes of the nanohydroxyapatite. These processes exert a significant influence on nanopowder characteristics. Calcination indicates strong distortions of the nanoHAP lattice and reduces crystallinity, depending on the substituted ions, which leeds to particle aggregation and modifications in surface morphology. Diverse sizes and an irregular, finely textured morphology within the nanoHAP, hold considerable promise for promoting cell commitment and osteoconduction. Additionally, the formation of new phases in the bioceramic structure, such as β-TCP, CaO, and MgO, significantly impacts ion release levels and biological activity (osteoblast proliferation and differentiation) of the material. Altered crystallite size influences specific surface area and correlates with ion release. It suggests that both ion modification and thermal treatment strongly affects the nanopowders' properties.

The data presented in this study contribute to developing advanced biomaterials for application in bone regeneration and replacement. The results show that substitution of elements, especially Mg^2+^, Sr^2+^, make nanoHAP a multifunctional material ready for further applications or investigations as a component of more advanced biomaterials. In addition, calcination may additionally ameliorate the final properties of bioceramics. The synergistic effect achieved through the combination of calcination and ionic modifications presents new opportunities for designing customizable biomaterials with tailored properties, offering novel prospects for various biological and medical applications.

### Supplementary Information

Supplementary Figure S1.

## Data Availability

The data generated during this study are available at ŁUKASIEWICZ Research Network Institute of Ceramics and Building Materials, Center of Ceramic and Concrete in Warsaw, Biomaterials Research Group, Postępu 9, Warsaw, 02-676, Poland. Biological research data are available at University of Lodz, Faculty of Biology and Environmental Protection, Department of Immunology and Infectious Biology, Banacha 12/16, 90-237 Lodz, Poland. All data are available from the corresponding author upon request.

## Abbreviations

HAP: Hydroxyapatite
nanoHAP: Nanohydroxyapatite
β-TCP: β-Tricalcium phosphate
