# Supplementary material for: Calcination and ion substitution improve physicochemical and biological properties of nanohydroxyapatite for bone tissue engineering applications
Source: Sci Rep. 2023 Sep 16;13:15384. doi: 10.1038/s41598-023-42271-2 (PMC10505220; doi:10.1038/s41598-023-42271-2)
Supplement: Supplementary file 1 — Supplementary Figure S1. [file 41598_2023_42271_MOESM1_ESM.docx]

**Supplementary**


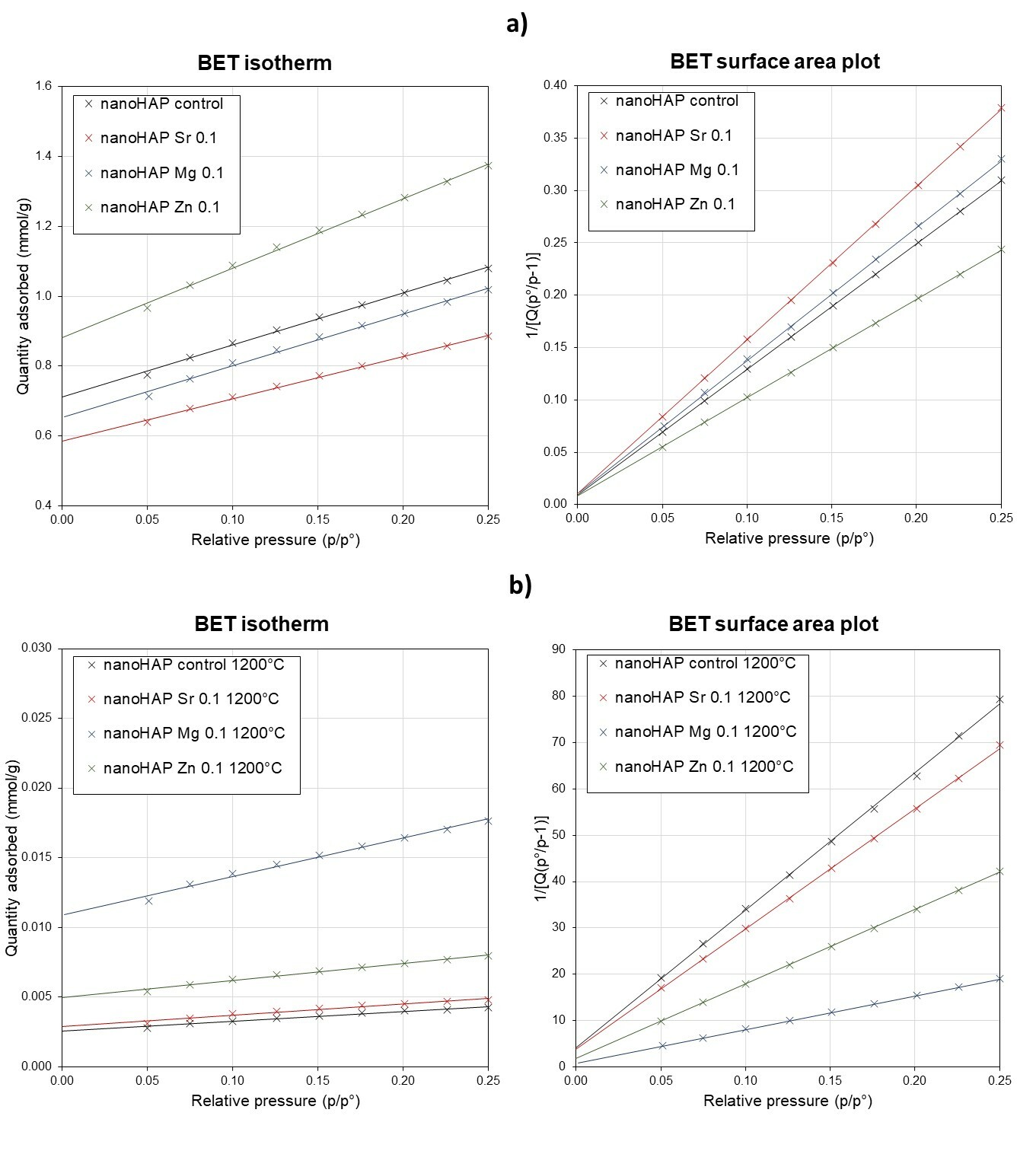
**Figure S1.** BET isotherms and BET surface area plots of materials a) before and b) after the calcination at 1200°C.
